# New Insights into Chloramphenicol Biosynthesis in Streptomyces venezuelae ATCC 10712

**DOI:** 10.1128/AAC.04272-14

**Published:** 2014-12

**Authors:** Lorena T. Fernández-Martínez, Chiara Borsetto, Juan Pablo Gomez-Escribano, Maureen J. Bibb, Mahmoud M. Al-Bassam, Govind Chandra, Mervyn J. Bibb

**Affiliations:** Department of Molecular Microbiology, John Innes Centre, Norwich Research Park, Norwich, United Kingdom

## Abstract

Comparative genome analysis revealed seven uncharacterized genes, *sven0909* to *sven0915*, adjacent to the previously identified chloramphenicol biosynthetic gene cluster (*sven0916–sven0928*) of Streptomyces venezuelae strain ATCC 10712 that was absent in a closely related Streptomyces strain that does not produce chloramphenicol. Transcriptional analysis suggested that three of these genes might be involved in chloramphenicol production, a prediction confirmed by the construction of deletion mutants. These three genes encode a cluster-associated transcriptional activator (Sven0913), a phosphopantetheinyl transferase (Sven0914), and a Na^+^/H^+^ antiporter (Sven0915). Bioinformatic analysis also revealed the presence of a previously undetected gene, *sven0925*, embedded within the chloramphenicol biosynthetic gene cluster that appears to encode an acyl carrier protein, bringing the number of new genes likely to be involved in chloramphenicol production to four. Microarray experiments and synteny comparisons also suggest that *sven0929* is part of the biosynthetic gene cluster. This has allowed us to propose an updated and revised version of the chloramphenicol biosynthetic pathway.

## INTRODUCTION

Chloramphenicol (CHL) is a bacteriostatic antibiotic that inhibits protein synthesis by binding to the 50S subunit of the bacterial ribosome. It is active against both Gram-negative and Gram-positive bacteria, including many multiply drug-resistant strains. CHL is produced by several Gram-positive soil actinomycetes, but its biosynthesis has been analyzed mostly in Streptomyces venezuelae strain ATCC 10712 (1). The initial stages of CHL biosynthesis utilize the shikimate pathway that leads to the production of chorismic acid. Chorismic acid serves as a branch point for aromatic amino acid (phenylalanine, tyrosine, and tryptophan) biosynthesis and for the production of *p*-aminobenzoic acid (PABA) that is required for folic acid biosynthesis (2). The conversion of chorismic acid to PABA occurs via 4-amino-4-deoxychorismate the precursor for the pathway dedicated to chloramphenicol biosynthesis (3).

Previous analyses of the CHL biosynthetic gene cluster of S. venezuelae resulted in the identification of 12 genes with a proven or likely role in CHL production (3–10). For reasons that we do not understand, we were unable to detect CHL production by S. venezuelae under our laboratory conditions, but we did succeed in expressing the CHL biosynthetic gene cluster from this strain in the heterologous host Streptomyces coelicolor A3 (2, 11).

Bioinformatic analysis has become a powerful tool for the identification of natural product biosynthetic gene clusters (12, 13). By combining comparative genomics with microarray and bioinformatic analyses, we have identified four additional genes likely to be involved in CHL production, and we confirmed the participation of three of these in antibiotic biosynthesis by mutational analyses.

## MATERIALS AND METHODS

### Strains and general methods.

The strains used and generated in this study are listed in Table 1. Escherichia coli strains were grown and manipulated according to standard methods (14–16). S. coelicolor and S. venezuelae strains were grown as described previously on soya flour mannitol (SFM) or DNA agar medium (17) and in glucose-yeast extract-malt extract (GYM) (18) or in maltose-yeast extract-malt extract (MYM) (19) liquid medium. The plasmids and oligonucleotides used are described in Table 2 and Table S1 in the supplemental material, respectively.

**TABLE 1:** Strains used and constructed in this study

Strain	Genotype	Reference or source
E. coli BW25113	K-12 derivative (Δ*araBAD* Δ*rhaBAD*) carrying plasmid pIJ790	40
E. coli DH5α	F^−^ ϕ80d*lacZ*ΔM15 Δ(*lacZYA-argF*) *U169 recA1 endA1 hsdR17* (r_K_^−^ m_K_^+^) *phoA supE44 thi-1 gyrA96 relA1* λ^−^)	41
E. coli ET12567	*dam-13*:: Tn*9 dcm-6 hsdM* CHL^r^, carrying helper plasmid pUZ8002	42
E. coli TOP10	DH10B derivative	Invitrogen
M. luteus ATCC 4698	Bioassay indicator microorganism	ATCC
S. coelicolor M1152	M145 derivative Δ*act* Δ*red* Δ*cpk* Δ*cda rpoB*(C1298T)	11
S. coelicolor M1581	M1152 containing cosmid pAH91 (S. venezuelae cosmid containing CHL cluster)	This work
S. coelicolor M1583	M1152 pAH91 (Δ*sven0909*–Δ*sven0912*)	This work
S. coelicolor M1584	M1152 pAH91 (Δ*sven0913*–Δ*sven0915*)	This work
S. coelicolor M1585	M1152 pAH91 (Δ*sven0913*)	This work
S. coelicolor M1586	M1152 pAH91 (Δ*sven0914*)	This work
S. coelicolor M1587	M1152 pAH91 (Δ*sven0915*)	This work
S. coelicolor M1588	M1152 pAH91 (Δ*sven0913*) + pIJ12745	This work
S. coelicolor M1589	M1152 pAH91 (Δ*sven0914*) + pIJ12747	This work
S. coelicolor M1590	M1152 pAH91 (Δ*sven0915*) + pIJ12748	This work
S. venezuelae ATCC 10712	Wild-type S. venezuelae strain	ATCC
S. venezuelae M1582	S. venezuelae with pIJ12744 (ermE*p::*sven0915*)	This work
S. venezuelae M1591	S. venezuelae with pIJ10257	This work

**TABLE 2:** Vectors and constructs used in this study

Vector/construct	Description^*a*^	Reference or source
pAH91	Conjugative and integrative (ϕC31 *attB*) derivative of cosmid 4P22 containing the CHL gene cluster from S. venezuelae, APR^r^ CAR^r^	11
pIJ10700	pBluescript II KS(+) containing *hyg-oriT* cassette	16
pUZ8002	*tra*, *neo*, RP4	43
pIJ790	λ-RED (*gam*, *bet*, *exo*), *cat*, *araC*, *rep101^ts^*	44
pR9406	Driver plasmid, CAR^r^ derived from pUB307	45; David Figurski, personal communication
pIJ10257	*oriT*, ϕBT1 *attB-int*, HYG^r^, ermE*p	22
pIJ12744	pIJ10257 with *sven0913*, HYG^r^	This work
pRT802	Conjugative and ϕBT1-integrative vector, KAN^r^	20
pIJ12551	Conjugative and ϕC31-integrative vector, APR^r^	21
pIJ12744	pIJ10257 with *sven0913* under ermE*p control, HYG^r^	This work
pIJ12745	pRT802 with *sven0913* and its promoter region, KAN^r^	This work
pIJ12746	pIJ12551 with *sven0914* under ermE*p control, APR^r^	This work
pIJ12747	pRT802 with *sven0914* under ermE*p control, KAN^r^	This work
pIJ12748	pRT802 with *sven0915* and its promoter region, KAN^r^	This work

a APR^r^, apramycin resistance; CAR^r^, carbenicillin resistance; HYG^r^, hygromycin resistance; KAN^r^, kanamycin resistance.

### Construction of deletion mutants.

Genes carried by pAH91 (Andrew Hesketh, personal communication; Gomez-Escribano and Bibb [11]) were replaced either individually (*sven0913*, *sven0914*, and *sven0915*) or in groups (*sven0909* to *sven0912* and *sven0913–sven0915*) with a hygromycin (HYG) resistance cassette amplified from pIJ10700 using the primer pairs listed in Table S1 in the supplemental material, as described by Gust et al. (15). The mutations were confirmed by PCR using flanking primers. Conjugations between E. coli strain ET12567 with plasmid pUZ8002 carrying the *oriT*-containing pAH91 derivatives and streptomycete strains were carried out as described previously (17).

### Complementation of deletion mutants.

To complement the *sven0913*, *sven0914*, and *sven0915* deletion mutants, PCR products were generated by high-fidelity PCR using the primers listed in Table S1 in the supplemental material. For the complementation of Δ*sven0913* and Δ*sven0915*, the PCR products extended from the beginning of the upstream intergenic region to the stop codon of each gene. The fragments were cloned into pRT802 (20) to generate pIJ12745 and pIJ12748, respectively, which were then transferred into the corresponding mutant strains using E. coli strain DH10B transformed with either of the plasmids in triparental matings with E. coli TOP10 (Invitrogen) containing the driver plasmid pR9406 and the nonmethylating E. coli strain ET12567, according to standard procedures (17). For the complementation of Δ*sven0914*, the coding sequence between the start and stop codons of the gene was amplified and cloned into pIJ12551 (21) to fuse *sven0914* to the constitutive ermE* promoter generating pIJ12746. A BamHI fragment from pIJ12746 containing ermE*p-*sven0914* was then cloned into pRT802 to generate pIJ12747, which was manipulated as above.

### Overexpression of *sven0913*.

A PCR product containing *sven0913* (extending from the start to stop codons) was generated by high-fidelity PCR using the primers listed in Table S1 in the supplemental material. This fragment was cloned into the integrative vector pIJ10257 (22) to fuse *sven0913* to the constitutive ermE* promoter, generating pIJ12744. The vector was transferred into S. venezuelae by conjugation from E. coli ET12567(pUZ8002), as described previously (17).

### HPLC analysis.

Chloramphenicol production was quantified by high-performance liquid chromatography (HPLC). The culture supernatants were filtered through VectaSpin Micro polysulfone 0.2-mm columns (Whatman, Maidstone, United Kingdom), injected onto a Spherisorb 5-mm ODS2 4.6 by 250 mm C_18_ column (Waters, Milford, MA, USA) fitted to an Agilent 1100 HPLC system with a diode array detector and analyzed using a method modified from He et al. (4; A. Hesketh, personal communication): gradient water-methanol; min 0, 0% methanol; min 2, 25% methanol; min 12, 50% methanol, min 14, 100% methanol; min 20, 100% methanol; and min 22, 0% methanol. CHL eluted at about 15.8 min and was detected at 273 nm. CHL (catalog no. C03478; Sigma) was used as a standard.

### DNA microarray analysis.

RNA isolation from S. venezuelae and subsequent DNA microarray analysis were carried out as described previously (23).

### Real-time PCR analysis.

Streptomyces strains were cultured in liquid GYM medium in triplicate, as described previously (17). RNA was extracted according to published procedures (24) from 2.5 ml of culture sampled after 16 h of growth of the S. venezuelae strains and after 48 h of growth of the S. coelicolor M1152 derivatives. Mycelial pellets were resuspended in 1 ml of RTL buffer with lysing matrix B (MP Biomedicals) and homogenized using a FastPrep instrument (BIO 101). Two pulses of 30 s of intensity 6.0 were applied, with cooling down for 1 min on ice between pulses. The supernatants were centrifuged for 10 min at 13,000 rpm and then treated according to the instructions given in the RNeasy kit (Qiagen, Crawley, United Kingdom). The RNA samples were treated with DNase I (Invitrogen) until they were free of DNA contamination. The RNA was quantified, and equal amounts from each sample were converted to cDNA, according to the manufacturer's instructions (SuperScript; Invitrogen). The oligonucleotide pairs listed in Table S1 in the supplemental material were used to amplify the genes representing each of the putative operons within the CHL biosynthetic cluster, as well as the left and right flanking genes (*sven0912* and *sven0930*, respectively). Amplification was also attempted using the same oligonucleotide pairs on RNA samples that had not been treated with reverse transcriptase to confirm a lack of DNA contamination.

## RESULTS

### Comparative genome and microarray analyses reveal three putative new members of the chloramphenicol gene cluster.

**FIG 1:**
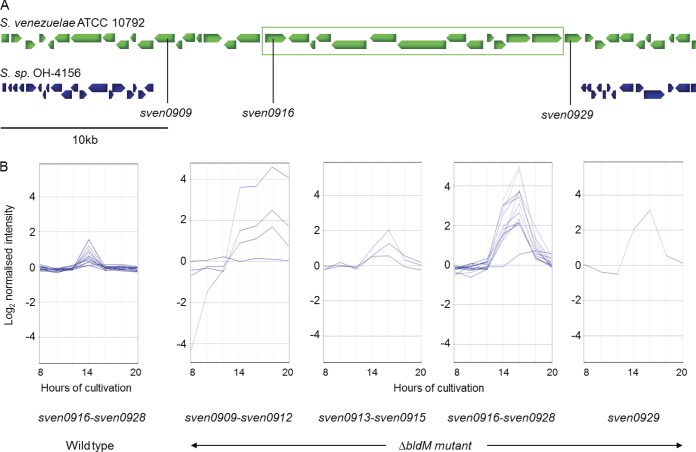
(A) Alignment of Solexa contigs (shown as filled blue arrows) from the draft genome sequence of Streptomyces sp. strain OH-4156 with the region of the S. venezuelae genome encoding CHL biosynthesis (protein-coding sequences are shown as filled green arrows). A Solexa contig was aligned to the S. venezuelae genome sequence if it exhibited ≥80% identity over a length of ≥100 nucleotides (average level of nucleotide sequence identity between the Solexa contigs and the S. venezuelae genome sequence was 88.5%, with a modal value of 93%). The alignment shows a cluster of 21 genes (*sven0909–sven0929*) unique to S. venezuelae that includes the 12 previously implicated in CHL biosynthesis (*sven0916–sven0928*, shown in the green rectangle). (B) Microarray expression profiles of the 21 genes suggested that *sven0913–sven0929* may be transcriptionally coregulated. The *x* axis represents culture age, and the *y* axis is normalized transcript abundance on a log_2_ scale.

Comparative genome analysis can play an extremely useful role in identifying and determining the extent of natural product biosynthetic gene clusters. The genome mining of Streptomyces sp. strain OH-4156 (25), which does not produce CHL, indicated a high level of nucleotide sequence similarity with the genome of S. venezuelae. The alignment of the Streptomyces sp. strain OH-4156 Solexa contigs on the S. venezuelae genome sequence in the region of the CHL biosynthetic gene cluster revealed the presence of seven genes (*sven0909–sven0915*) adjacent to the previously identified CHL biosynthetic gene cluster (*sven0916–sven0928*) that were absent in the nonproducing strain (Fig. 1A). To gain insight into whether any of these newly identified genes might play a role in CHL production, we compared their transcriptional profiles with those of established CHL biosynthetic genes in the microarray data from submerged cultures of S. venezuelae and several of its developmental mutants that are deficient in sporulation. Although we were unable to detect CHL biosynthesis in S. venezuelae, the transcription of *sven0916–sven0928* was detected in the wild-type strain at a low level, peaking at 14 h of cultivation (Fig. 1B). Surprisingly, the transcription of these genes was markedly increased in a *bldM* mutant and showed a pattern of expression similar to that in the wild-type strain, but peaking after 16 h of cultivation (Fig. 1B). Analysis of the expression profiles for *sven0909–sven0915* suggested that three of these genes, *sven0913–sven0915*, might be coordinately regulated with *sven0916–sven0928*, genes known or believed to be involved in chloramphenicol biosynthesis, and which thus might form part of an extended CHL biosynthetic gene cluster (Fig. 1B). These three genes were predicted to encode a transcriptional activator (*sven0913*) with 44% amino acid sequence identity to StrR, the pathway-specific activator of the streptomycin biosynthetic gene cluster in Streptomyces griseus, a phosphopantetheinyl transferase (PPTase) (*sven0914*), and a Na^+^/H^+^ antiporter (*sven0913*). In contrast, the expression patterns of *sven0909–sven0912*, all encoding hypothetical proteins, were different from those of the known CHL biosynthetic genes, suggesting that they may not be part of the CHL cluster (Fig. 1B).

### Deletion of *sven0913–sven0915* abolishes CHL production.

**FIG 2:**
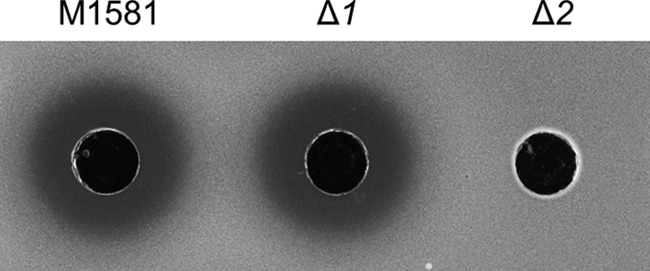
Analysis of the effect on CHL biosynthesis of replacing *sven0909–sven0912* or *sven0913–sven0915* with the *hyg* cassette from pIJ10700. S. coelicolor M1581 and its mutant derivatives M1583 (Δ*1* for *sven0909–sven0912*) and M1584 (Δ*2* for *sven0913–sven0915*) were grown for 6 days in GYM liquid medium, and 100 μl of culture supernatant was assayed for antibiotic activity against M. luteus. The absence of an inhibition zone in the Δ*2* mutant indicates an essential role for at least one of the *sven0913–sven0915* genes in CHL biosynthesis.

To assess whether *sven0909–sven0912* and *sven0913–sven0915* play a role in CHL biosynthesis, each set of genes was deleted from the cloned CHL biosynthetic gene cluster of pAH91 by PCR targeting. The HYG resistance cassette from pIJ10700 (15) was used to replace *sven0909–sven0912* (Δ*sven0909*–Δ*sven0912*) and *sven0913–sven0915* (Δ*sven0913*–Δ*sven0915*) in E. coli, and the mutated cosmids were transferred to S. coelicolor M1152 by conjugation, yielding M1583 (Δ*sven0909*–Δ*sven0912*) and M1584 (Δ*sven0913*–Δ*sven0915*), respectively. The integration of each cosmid was confirmed by PCR. The two strains, together with S. coelicolor M1152 carrying pAH91 (M1581), were grown in GYM liquid medium (18), and the supernatants from each culture were spotted onto filter paper discs laid on top of a lawn of Micrococcus luteus (Fig. 2). While the supernatant from M1583 (Δ*sven0909*–Δ*sven0912*, Δ*1* in Fig. 2) produced a zone of inhibition identical to that of M1581, that from M1584 (Δ*sven0913*–Δ*sven0915*, Δ*2* in Fig. 2) showed no inhibitory activity, and HPLC analysis confirmed the absence of CHL in the supernatant (data not shown), indicating that at least one of the genes *sven0913–sven0915* plays an essential role in CHL biosynthesis.

### Individual deletion of *sven0913*, *sven0914*, and *sven0915* confirms their role in CHL production.

**FIG 3:**
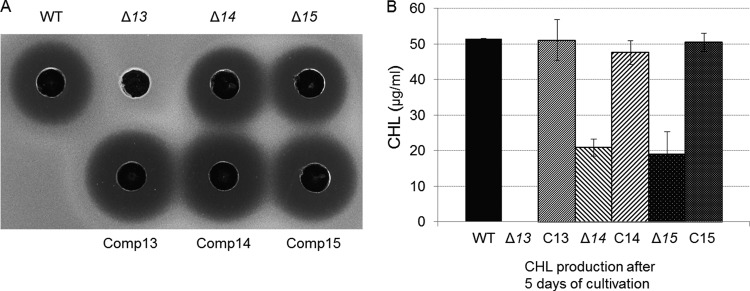
Analysis of the effects of single-gene deletions on CHL production. S. coelicolor M1581 and its mutant derivatives M1585 (Δ*sven0913* [Δ*13*]), M1586 (Δ*sven0914* [Δ*14*]), and M1587 (Δ*sven0915* [Δ*15*]) and their complemented derivatives (Comp13, Comp14, and Comp15, respectively) were grown for 5 days in GYM liquid medium. (A) One hundred microliters of supernatant from each of the cultures was assayed for antibiotic activity against M. luteus. (B) Quantitation of CHL production by each of the strains by HPLC analysis. Bars indicate the standard deviation for three biological samples.

To investigate the individual roles of *sven0913–sven0915* in CHL biosynthesis, PCR targeting was carried out on pAH91 to separately delete each gene. The deletion of s*ven0913* (yielding M1585), the putative transcriptional activator, abolished antibiotic activity (Fig. 3A) and CHL production (Fig. 3B). In contrast, the deletion of either *sven0914* (to give M1586), coding for a putative PPTase, or *sven0915* (yielding M1587), encoding a putative Na^+^/H^+^ antiporter, decreased the level of CHL production to around 40% of that observed with the wild-type gene cluster. Presumably, the roles that Sven0914 and Sven0915 play in CHL biosynthesis can be at least partially substituted by proteins encoded elsewhere in the S. coelicolor genome.

The complementation of each of the three mutant strains was accomplished using integrative vectors containing the gene of interest expressed from either its native promoter (*sven0913* and *sven0915*) or from the constitutive ermE* promoter (*sven0914*). In each case, CHL production was restored to the level observed with the wild-type gene cluster (Fig. 3).

### Constitutive expression of *sven0913* activates CHL production in S. venezuelae.

**FIG 4:**
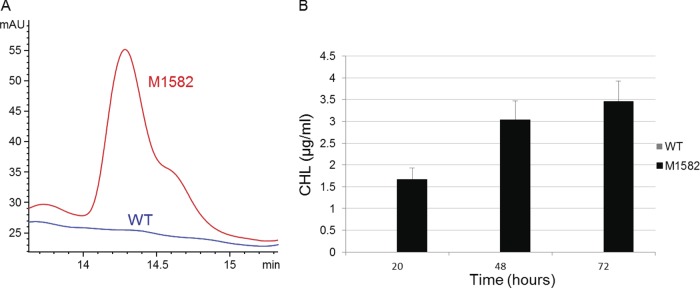
CHL production in wild-type (WT) S. venezuelae and in M1582 (with *sven0913* expressed from the ermE* promoter). (A) HPLC chromatogram of CHL present in supernatants of 72 h cultures measured at a wavelength of 273 nm (see Materials and Methods). (B) CHL production, estimated as in panel A by HPLC analysis, after 20, 48, and 72 h of culture. Bars indicate the standard deviation for three biological samples.

As mentioned previously, we were unable to detect chloramphenicol production in S. venezuelae under a variety of growth conditions (Fig. 4). Since our results suggest that *sven0913* likely acts as a transcriptional activator of the CHL biosynthetic gene cluster, we attempted to overexpress this gene in S. venezuelae. To achieve this, the *sven0913* coding sequence was cloned in the integrative vector pIJ10257 (22) under the control of the constitutive ermE* promoter, and the resulting plasmid was transferred to S. venezuelae by conjugation and integration confirmed by PCR, yielding M1582. The supernatants obtained from this strain and from wild-type S. venezuelae were analyzed by HPLC for CHL production (Fig. 4). The constitutive expression of *sven0913* led to CHL production levels of approximately 3.5 μg/ml after 72 h of growth of M1582, while CHL biosynthesis remained undetectable in the vector-only control strain (M1591), consistent with the proposed role of *sven0913* as a cluster-situated transcriptional activator of the CHL biosynthetic gene cluster.

### RT-PCR analyses confirm that Sven0913 is a transcriptional activator of the CHL biosynthetic cluster.

**FIG 5:**
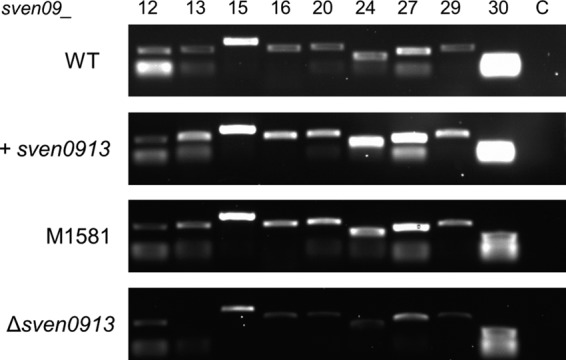
RT-PCR analysis of selected genes from the CHL biosynthetic gene cluster. RNA was isolated from 16 h cultures of the S. venezuelae strains and from 5-day cultures of the S. coelicolor M1152 derivatives and subjected to RT-PCR analysis. Top, wild-type S. venezuelae and M1582 (with *sven0913* expressed from the ermE* promoter [+*sven0913*]). Bottom, S. coelicolor M1581 and M1585 (with *sven0913* deleted [Δ*sven0913*]). The primer pairs listed in Table S1 in the supplemental material were used. The numbers at the top refer to the last two digits of the individual *sven* genes. A control (C) without reverse transcriptase and using primers corresponding to *sven0930* was used to confirm the absence of DNA contamination.

To confirm that *sven0913* plays a role in activating transcription of the CHL biosynthetic gene cluster, real-time PCR (RT-PCR) analysis was performed on the following strains: wild-type S. venezuelae, M1582 (S. venezuelae with constitutive expression of *sven0913*), M1581 (S. coelicolor M1152 carrying pAH91), and M1585 (M1581 with *sven0913* deleted). RNA was extracted from each of the cultures and RT-PCR conducted using primers amplifying the transcripts from several regions of the CHL gene cluster as well as from flanking genes (see Table S1 in the supplemental material). While the constitutive expression of *sven0913* in S. venezuelae resulted in elevated levels of expression of the genes predicted to lie within the CHL gene cluster, it had no effect on the flanking genes *sven0912* and *sven0930* (Fig. 5). Conversely, the deletion of *sven0913* in S. coelicolor (M1585) reduced the levels of expression of the predicted CHL biosynthetic genes but had no effect on those of the flanking genes. These results confirm that Sven0913 is a previously unidentified transcriptional activator that plays a crucial role in the regulation of CHL biosynthesis.

### *sven0925*, a previously unidentified gene encoding a putative acyl carrier protein.

**FIG 6:**
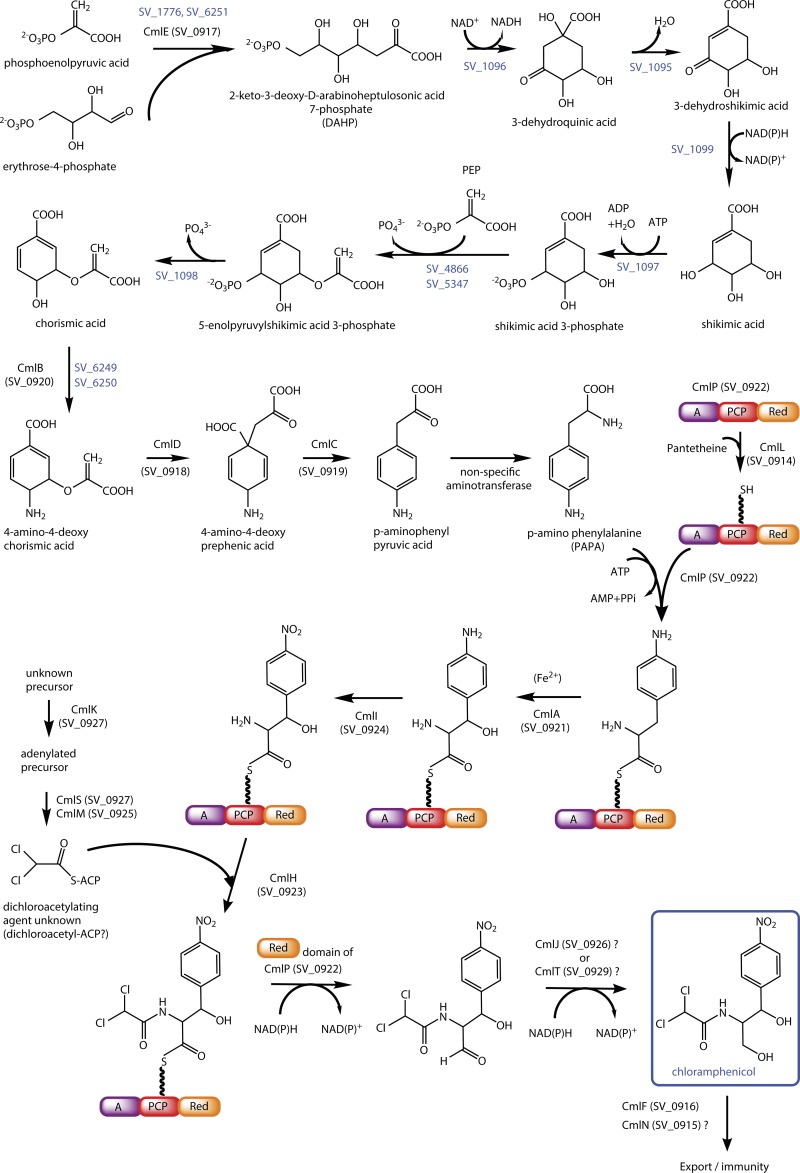
Revised pathway for CHL biosynthesis in S. venezuelae. SV_, protein designations from StrepDB (http://strepdb.streptomyces.org.uk/); those involved in the shikimate pathway and not part of the CHL gene cluster are shown in blue.

Our earlier annotation of the genome sequence of S. venezuelae (GenBank accession no. FR845719) had identified an additional previously undetected gene in the CHL biosynthetic gene cluster, *sven0925*, whose 108-amino-acid product failed to show clear similarity to the proteins of known function in the NCBI database. A more recent BLASTp search revealed a large number of homologues present in the genome sequences, some of which were annotated as acyl carrier proteins (ACPs). Based on this observation, Sven0925 was subjected to analysis using the Phyre2 protein structure prediction server (26) (http://www.sbg.bio.ic.ac.uk/phyre2). The program predicts with 97% to 98% confidence that Sven0925 is a homologue of structurally characterized ACPs from a variety of bacterial species; for example, 77 residues of Sven0925 (71% of its sequence) were modeled with 98.0% confidence to the structure of the holo-acyl carrier protein ne2163 from Nitrosomonas europaea (see Fig. S1 in the supplemental material). Moreover, Sven0925 possesses a conserved serine at position 50 that is predicted to be the catalytic residue needed for the addition of the 4′-phosphopantetheine moiety required for the activity of the holo-ACP (see Fig. S2 in the supplemental material). Together with the expression profile of *sven0925*, which conforms to that of the other CHL biosynthetic genes, we conclude that Sven0925 is indeed an ACP involved in CHL biosynthesis (see Fig. 6).

### *sven0929*, part of the CHL biosynthetic gene cluster, but not required for CHL production.

Synteny comparisons of *sven0900–sven0940* with the draft genome sequences of both Streptomyces sp. strain OH-4156 (Fig. 1A) and Micromonospora carbonacea strain ATCC 39149 (see below; see also Fig. S3 in the supplemental material) suggested that *sven0929* might be part of the CHL biosynthetic gene cluster. This inference was further supported by the microarray analysis (Fig. 1B) and RT-PCR experiments (Fig. 5), which clearly demonstrate that *sven0929* is transcriptionally coregulated with genes involved in CHL biosynthesis. However, the previous deletion of *sven0929* (ORF13 of Piraee, White, and Vining [9]), which appears to encode an aldo-keto reductase, had no apparent effect on CHL biosynthesis. We believe that *sven0929* is indeed part of the CHL biosynthetic gene cluster and that its role in CHL biosynthesis, like that of *sven0914* and *sven0915* in S. coelicolor, can be fulfilled by a functional homologue located elsewhere in the S. venezuelae genome.

## DISCUSSION

New developments in next-generation sequencing combined with bioinformatic analysis greatly facilitate the comparison of genome sequences of related bacteria. We have taken advantage of these advances and combined them with microarray analysis to identify four new members of the CHL biosynthetic gene cluster: *sven0913*, encoding a transcriptional activator; *sven0914*, encoding a putative PPTase; *sven0915*, encoding a putative ion antiporter; and *sven0925*, encoding a putative ACP.

For reasons we do not understand, while the transcription of the CHL biosynthetic gene cluster can be detected by microarray analysis in our wild-type isolate of S. venezuelae (Fig. 1A), albeit at a low level, we were unable to detect CHL production, despite using a range of culture media. However, the constitutive expression of *sven0913* resulted in readily detectable levels of antibiotic production, further highlighting the value of ectopically expressing cluster-situated regulatory genes to activate natural product biosynthesis (for an example, see reference 27).

The deletion of either *sven0914* or *sven0915*, which appear to be cotranscribed, decreased CHL production by approximately 60%. Production was restored to wild-type levels when each mutant strain was complemented with the respective gene, demonstrating that the phenotype of the Δ*sven0914* mutant is not simply the result of a polar effect of the deletion of *sven0915* and that both genes play a role in CHL production. Sven0914 is predicted to encode a PPTase. PPTases are responsible for the conversion of the inactive apo form of an ACP or peptidyl carrier protein (PCP) to the active holo form by covalent attachment of a coenzyme-A-derived phosphopantetheine group to a specific serine residue (28). The posttranslational phosphopantetheinylation of the apo-ACP/PCP domains is essential for the activities of many multienzyme synthases and synthetases responsible for the generation of a variety of natural products, most notably polyketides and nonribosomally synthesized peptides (29). Sven0914 contains all of the conserved motifs found in the F/KES subfamily of Sfp-type PPTases (30, 31). While many members of this subfamily utilize PCPs as substrates, there are exceptions, most notably the Sco6673-like PPTase of Streptomyces ambofaciens, which is able to accept both ACP and PCP domains as substrates (32) and which shares 53% amino acid sequence identity with Sven0914. Consequently, we cannot reliably predict whether Sven0914 is involved in the modification of the PCP domain of Sven0922 (see below), the ACP Sven0929, or both.

Interestingly, not all natural products that require the activity of a PPTase for their synthesis contain the corresponding gene within their biosynthetic gene clusters, and instead, they utilize a PPTase encoded elsewhere in the genome. Consequently, it is not too surprising that the deletion of *sven0914* reduced but did not abolish CHL production. Indeed, the CHL biosynthetic gene cluster of M. carbonacea ATCC 39149 (33) (GenBank accession no. GG657738) lacks a *sven0914* homologue, although all of the other genes required for CHL production are present and arranged in precisely the same manner as in S. venezuelae (see Fig. S3 in the supplemental material). A BLASTp search to find the possible homologues of Sven0914 in the heterologous host S. coelicolor identified Sco6673, with 54% amino acid sequence identity. A Pfam database search (34) identified a PPTase domain within the C-terminal region of Sco6673 and may explain why the deletion of *sven0914* still results in some CHL production in the heterologous host (the Sco6673 and the Sco6673-like proteins of S. ambofaciens share 83% identity).

*sven0915* is predicted to encode an Na^+^/H^+^ ion antiporter. Divergently transcribed from *sven0915* is *sven0916*, which is predicted to encode an efflux permease from the major facilitator superfamily, and it is presumably involved in CHL export. These efflux pumps are often involved in multidrug resistance, exporting toxic compounds to the outside of the cell, and they are usually driven by the energy stored in ion gradients to catalyze the transport of drugs across the membrane. Given their proximity in the CHL gene cluster, it is conceivable that *sven0915* provides the energy required for *sven0916* to export CHL outside the cell. The deletion of *sven0915* reduced CHL production by >60% in the heterologous host S. coelicolor. Several homologues of *sven0915* occur in the S. coelicolor M1152 genome, any one or more of which may partially compensate for the loss of Sven0915, thus allowing continued CHL export, although at lower levels, in the deletion mutant.

Our studies have identified four new genes that play, or that are likely to play, a role in CHL biosynthesis in S. venezuelae, prompting us to present an updated view of the CHL biosynthetic pathway (Fig. 6 and Table 3). Although previous studies have sometimes used different nomenclatures to represent the same gene, we have adopted that of Piraee, White, and Vining (9) to denote previously identified CHL biosynthetic genes, and we propose five new *cml* designations: *cmlR*, *cmlL*, *cmlN*, and *cmlM* for the newly identified genes described in this paper and *cmlT* for *sven0929*.

**TABLE 3:** Genes involved in CHL biosynthesis

*sven* no. (StrepDB)	Proposed nomenclature^*b*^	Function of gene product	Reference (of function)
*sven0913*^*a*^	*cmlR*	Transcriptional activator	This work
*sven0914*	*cmlL*	Phosphopantetheinyl transferase	Cosmid annotation/BLASTp
*sven0915*	*cmlN*	Integral membrane ion antiporter	Cosmid annotation/BLASTp
*sven0916*	*cmlF*	Chloramphenicol efflux pump	4, 9
*sven0917*	*cmlE*	DAHP synthase	4
*sven0918*	*cmlD*	4-Amino-4-deoxychorismate mutase	46
*sven0919*^*a*^	*cmlC*	4-Amino-4-deoxyprephenate dehydrogenase	46
*sven0920*^*a*^	*cmlB*	4-Amino-4-deoxychorismate synthase	46
*sven0921*	*cmlA*	Nonheme iron monooxygenase catalyzing β-hydroxylation of l-PAPA^*c*^	47
*sven0922*^*a*^	*cmlP*	Adenylation, PCP and reductase domains	4, 9
*sven0923*^*a*^	*cmlH*	Amidase	BLASTp; 4, 9
*sven0924*	*cmlI*	*N*-Oxygenase, nonheme diiron oxygenase.	10
*sven0925*	*cmlM*	Putative acyl carrier protein	Phyre^2^; 26
*sven0926*^*a*^	*cmlJ*	Short-chain dehydrogenase	4
*sven0927*	*cmlK*	Acyl-CoA-ACP synthetase, AMP-ligase	9
*sven0928*	*cmlS*	Flavin-dependent halogenase (chlorination of chloramphenicol)	9, 48
*sven0929*	*cmlT*	Aldo-keto reductase; not essential for CHL biosynthesis	9

a Deletion of that particular gene abolishes CHL production.

b Previous or alternative names given in the literature: *cmlD*, *papB* (46); *cmlC*, *papC* (46); *cmlB*, *pabAB* (4, 6), *papA* (46); *cmlP*, *cmlH* (GenBank accession no. AAG21975.2); *cmlH*, *cmlG* (8) (based on GenBank accession no. AAG21974.1).

c l-PAPA, l-*p*-aminophenylalanine; CoA, coenzyme A.

As previously noted, CHL biosynthesis utilizes the shikimate pathway, which leads to chorismic acid and hence aromatic amino acid production. Some of the chorismic acid is converted to 4-amino-4-deoxychorismate, which is used for synthesis of the essential metabolite PABA. 4-Amino-4-deoxychorismate is also utilized as the precursor for the pathway dedicated to CHL production. BLASTp searches of the S. venezuelae proteome confirmed that genes encoding homologues of two of the primary metabolic enzymes involved in 4-amino-4-deoxychorismate biosynthesis, 2-keto-3-deoxy-d-arabino-heptulosonate-7-phosphate (DAHP) synthase and 4-amino-4-deoxychorismate synthase, were present in the *cml* gene cluster (*sven0917* [*cmlE*] and *sven0920* [previously *pabAB* {9}, *cmlB* here], respectively. Homologues of *sven0917* and *sven0920* are also located elsewhere in the S. venezuelae genome and are presumably involved in primary metabolism. DAHP synthesis is the first committed step of the shikimate pathway and is often subject to feedback inhibition or repression in other microorganisms (35). Our transcriptional profiling suggests that CHL biosynthesis occurs at the end of rapid vegetative growth in S. venezuelae, when proteolytic degradation of existing proteins may provide an intracellular amino acid pool for continued morphological development. Such a source of aromatic amino acids might act to inhibit the expression and/or activity of the primary metabolic DAHP synthase and thus restrict the precursor pool for CHL biosynthesis. We speculate that the existence of a gene, *cmlE*, within the CHL biosynthetic gene cluster that encodes a DHAP synthase homologue may serve to ensure sufficient flux through the shikimate pathway for sustained CHL biosynthesis. Similarly, the presence of a 4-amino-4-deoxychorismate synthase homologue, *cmlB*, in the CHL biosynthetic gene cluster may act to direct flux toward CHL biosynthesis rather than continued aromatic amino acid production.

The work reported in this paper extends previous studies that led to an increasingly informed understanding of CHL production in S. venezuelae (3–6, 9, 36), and it allows us to present a consolidated and updated view of the CHL biosynthetic pathway (Fig. 6 and Table 3). Although the functions of many genes can be assigned with certainty or a high level of confidence, the roles of those involved in the dichloroacetylation of the CHL precursor remain obscure. Partly by a process of elimination, these genes are likely to include *sven0925* (*cmlM*), *sven0926* (*cmlJ*), *sven0927* (*cmlK*), and *sven0929* (*cmlT*), as well as *sven0928* (*cmlS*), encoding a flavin-dependent halogenase. All five of these genes are clustered together and may well be contained in a single operon, consistent with a common function. Based on the similarity of CmlK to the adenylating enzymes PchD and DhbE, which utilize salicylic acid and 2,3-dihydroxybenzoic acid as substrates, respectively, Piraee, White, and Vining (9) speculated that dichloroacetylation occurs through CmlK-mediated adenylation of an aromatic carboxylic acid. The recognition of the putative ACP CmlM in this study suggests that CmlM may also be a substrate for CmlK, tethering the carboxylic acid prior to halogenation (Fig. 6).

Surprisingly, microarray analyses revealed that the expression of the CHL biosynthetic gene cluster was markedly enhanced in *bldM*, *whiI*, *bldN*, and *whiG* (data not shown) mutants of S. venezuelae. BldM and WhiI are both orphan atypical response regulators that play a crucial role in morphological differentiation. Recent work has demonstrated that BldM and WhiI can form a heterodimer that activates transcription from so-called group II promoters, presumably integrating signals from two distinct developmental pathways (37). Chromatin immunoprecipitation sequencing (ChIP-Seq) analysis by the same authors failed to reveal binding of either protein to any of the potential promoter regions in the CHL biosynthetic gene cluster, nor did we find a sequence motif corresponding to the consensus recognition sequence for group II promoters anywhere in the gene cluster. It thus seems unlikely that a BldM-WhiI heterodimer can directly repress CHL gene expression in the wild-type strain. The elevated levels of CHL gene transcription observed in both *bldM* and *whiI* mutants may thus reflect the existence of an as-yet-unidentified gene that negatively regulates CHL production and whose expression is dependent on the BldM-WhiI heterodimer. *bldM* is transcribed from two promoters, one of which (p1) is directly activated by BldN at the onset of development (23, 38), and thus the effect of deleting *bldN* on CHL gene transcription is presumably mediated through decreased levels of *bldM* transcription. Similarly, the transcription of *whiI* is activated from a single WhiG-dependent promoter (39), presumably explaining the effect of mutation of *whiG* on *cml* gene transcription.

## Supplementary Material

Supplemental material
