# Supplementary material for: New Insights into Chloramphenicol Biosynthesis in Streptomyces venezuelae ATCC 10712
Source: Antimicrob Agents Chemother. 2014 Dec;58(12):7441–50. doi: 10.1128/AAC.04272-14 (PMC4249514; doi:10.1128/AAC.04272-14)
Supplement: Supplemental material [file supp_58_12_7441__index.html]

New Insights into Chloramphenicol Biosynthesis in Streptomyces venezuelae ATCC 10712 — Supplemental material 

# New Insights into Chloramphenicol Biosynthesis in Streptomyces venezuelae ATCC 10712

## Supplemental material

**Files in this Data Supplement:**

- Supplemental file 1 -

  Supplemental Figures S1 to S3 and Table S1.

  PDF, 171K
