# Supplementary material for: New Insights into Chloramphenicol Biosynthesis in Streptomyces venezuelae ATCC 10712
Source: Antimicrob Agents Chemother. 2014 Dec;58(12):7441–50. doi: 10.1128/AAC.04272-14 (PMC4249514; doi:10.1128/AAC.04272-14)
Supplement: Supplemental material [file AAC.04272-14_zac012143510so1.pdf]

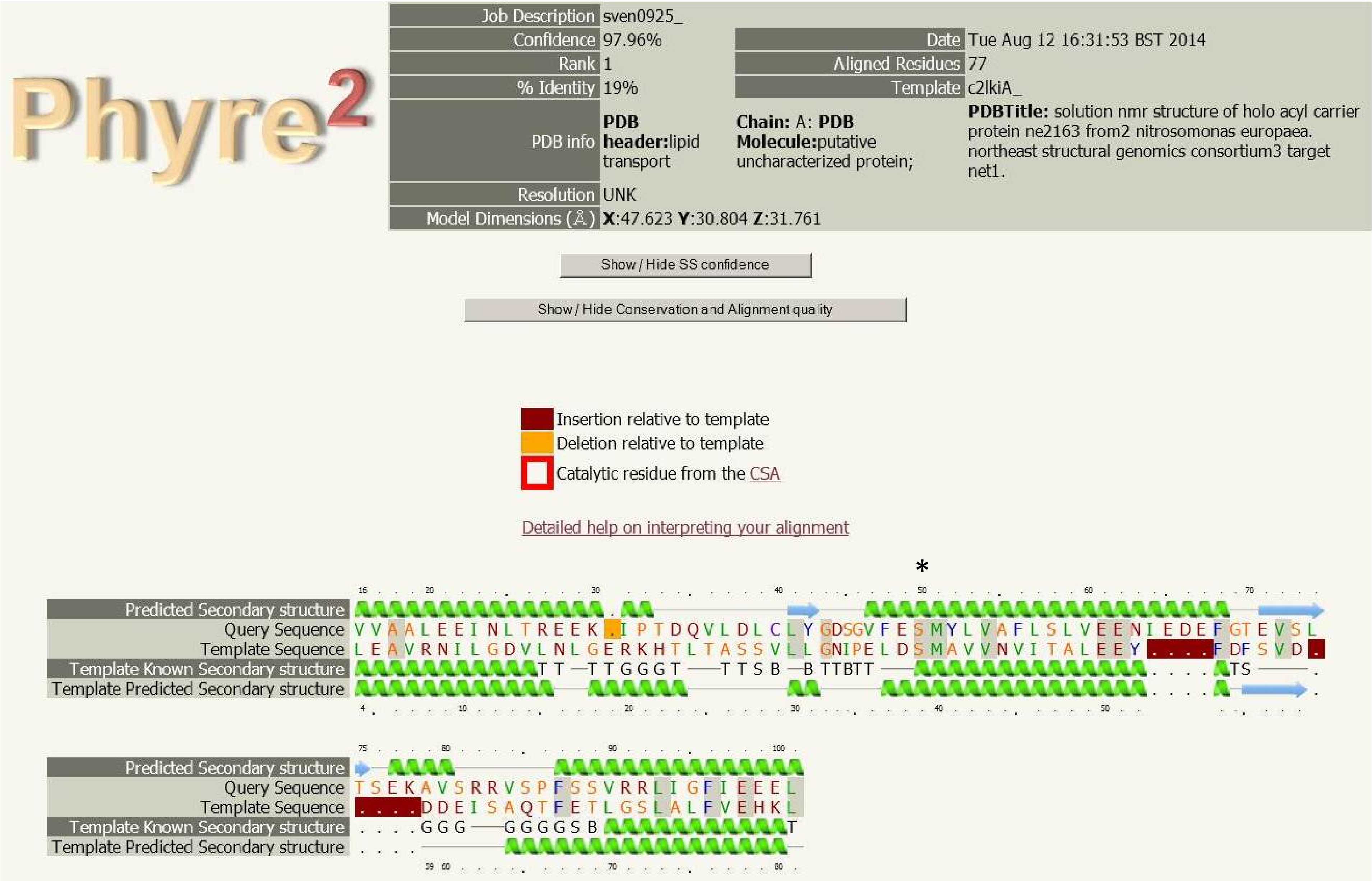

FIG. S1. Phyre[2] alignment of Sven0925 to the holo acyl carrier protein ne2163 from *Nitrosomonas europaea*. 77 residues of Sven0925 (71% of its sequence) were modelled with 98% confidence to the structure of the holo acyl carrier protein (output from Phyre2; http://www.sbg.bio.ic.ac.uk/phyre2). *, conserved serine at position 50 of Sven0925 that is predicted to be the catalytic residue needed for addition of the 4'-phosphopantetheine moiety .

```
Bacillus_cereus             ---------MEDTNKEKIIQVVLCCVEEVNENLENKIDLEKGLKAHLYDW 41
Bacillus_bombysepticus      ---------MEDTNKEKIIQIVLCCVEEVNENLENKIDLEKGLKAHLYDW 41
Bacillus_thuringiensis      MICKKECIYLEDTKKEKIIQIVLCCVEEVNENLENKIDLEKGLKAHLYDW 50
Pelosinus                   -------------MEQKIIGIILHEVSELNEQLDHKVAIENGENAGLYGL 37
Sven0925                    ------MLDGGLKVTERIQGVVVAALEEINLTREEKIPTDQVLDLCLYGD 44
Desulfovibrio               -----------MPLRQQVKQALIEDLN-LQSITPEDIEDDAP----LFG- 33
                                           :::   ::  :. ::    ..:  :      *:.

                                  #
Bacillus_cereus             EGY-LDSLGLVSLLVSIEQGLEDELGLSVDLVNLDTMSQHNSPFKTIEAL 90
Bacillus_bombysepticus      EGY-LDSLGLVSLLVSIEQGLEDELGLSVDLVNLDTMSQHNSPFKTIEAL 90
Bacillus_thuringiensis      EGY-LDSLGLVSLLVSIEQGLEDELGLSVDLVNLDTMSQHNSPFKTIETL 99
Pelosinus                   TGV-LDSLGLVTLIVAVEQAIEDELGLAISLADERAMSQKNSPFRTIGTL 86
Sven0925                    SGV-FESMYLVAFLSLVEENIEDEFGTEVSLTSEKAVSRRVSPFSSVRRL 93
Desulfovibrio               DGLGLDSLDAVELVVLVQK----RFGVEIKNMDEGRVA-----FASVTAL 74
                             *  ::*:  * ::  :::    .:*  :.  .   ::     * ::  *

Bacillus_cereus             VAYILECQEQLKSLT 105
Bacillus_bombysepticus      VAYILECQEQLKSLT 105
Bacillus_thuringiensis      VAYIIECQETLKSLT 114
Pelosinus                   ADYIVKVSQEEA--- 98
Sven0925                    IGFIEEELELVGVRG 108
Desulfovibrio               AGFIEERR------- 82
                              :* :
```

Percent amino acid sequence identity

	Sven0925
Desulfovibrio	34.15
Pelosinus fermentans	30.61
Bacillus thuringiensis	29.63
Bacillus cereus	31.43
Bacillus bombysepticus	30.48

FIG. S2. Clustal Omega alignment of Sven0925 to homologous proteins identified in a blastP search of the NCBI database as acyl carrier proteins. #, conserved serine at position 50 of Sven0925 that is predicted to be the site of addition of the 4'-phosphopantetheine moiety. Percentage amino acid sequence identities to Sven0925 are given below the alignment.

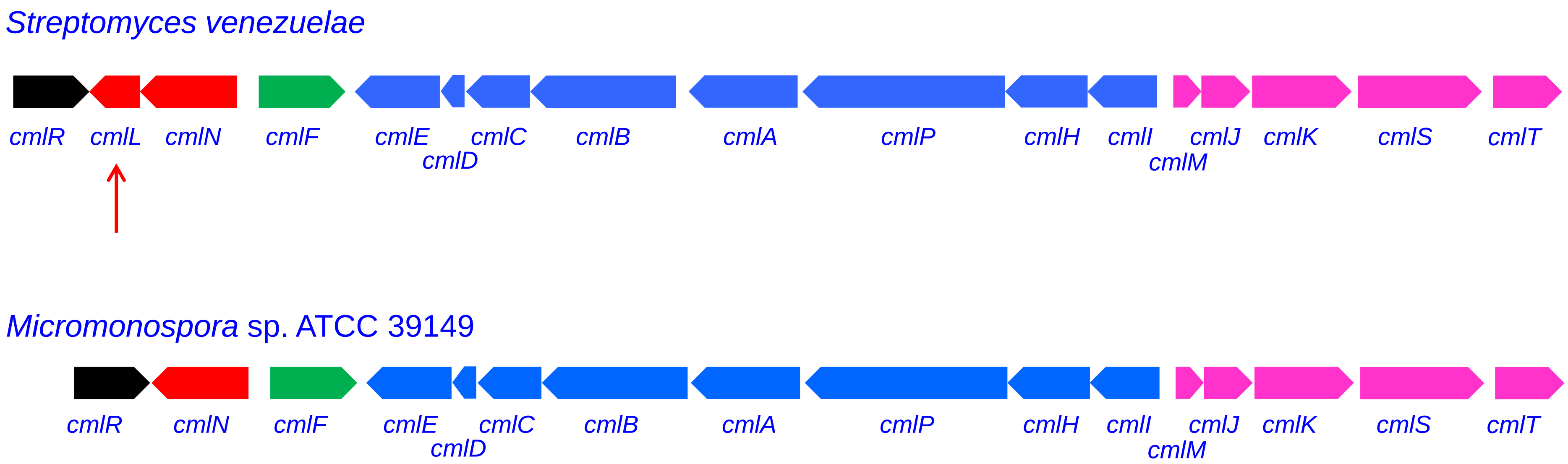

FIG. S3. Comparison of the CHL biosynthetic gene clusters of *S. venezuelae* and *Micromonospora* sp. ATCC 39149. Note the absence of a *cmlL* homologue in the *Micromonospora* gene cluster. The different colours represent potential transcription units.

Table S1. Oligonucleotides used in this study.

Name	Sequence	Notes
HygF0909_PCR_T1	TCAGTCGCCTCGGGCCGTGGCTGCCGCGTGG GCGGTCGGCTGACGCCGTTGGATACACC	HYG^R cassette for PCR-Targeting *sven0909- sven0912*
HygR0912_PCR_T1	TGCACGACGGCTGGCTCCGGAGCCGTACGGG GCACGGCGTCAGGCGCCGGGGGCGGTGT	HYG^R cassette for PCR-Targeting *sven0909- sven0913*
HygF0913_PCR_T2	ATGTCCACGATTTCGGATCTACGACAGGCGTC GACCTTCCTGACGCCGTTGGATACACC	HYG^R cassette for PCR-Targeting *sven0913- sven0915*
HygR0915_PCR_T2	ATGACGACCCACCAGACAGCCATGATCATGAT GGGGCTCTCAGGCGCCGGGGGCGGTGT	HYG^R cassette for PCR-Targeting *sven0913- sven0915*
Hyg0913_F_KO	CTTTCCTGTCATCGATGACGTGCGTTCCTGGAG GCATTGCTGACGCCGTTGGATACACC	HYG^R cassette for PCR-Targeting *sven0913*
Hyg0913_R_KO	GAGCGGGCGCCGCCGCTACGGCGGGGCGGC GGTAGGGGATCAGGCGCCGGGGGCGGTGT	HYG^R cassette for PCR-Targeting *sven0913*
Hyg0914_F_KO	GAAGCGAGCGGCGGCCGGACGCGGGCCCGG CCTGATCCCCTGACGCCGTTGGATACACC	HYG^R cassette for PCR-Targeting *sven0914*
Hyg0914_R_KO	CGCCCGCGCCGGCGGCGGGAAGCCGTCGGG GGCGAAGCGTCAGGCGCCGGGGGCGGTGT	HYG^R cassette for PCR-Targeting *sven0914*
Hyg0915_F_KO	GCGCCGCTTCCGGTACGAGCCCGGTCAGGAG CGTCGGCGCTGACGCCGTTGGATACACC	HYG^R cassette for PCR-Targeting *sven0915*
Hyg0915_R_KO	TAACGACTTGGACACCCCCGGCGCGGAGAAGG AGTGTTCTCAGGCGCCGGGGGCGGTGT	HYG^R cassette for PCR-Targeting *sven0915*
Compl13_SpeI_F	CCGTACTAGTCTGACCCGGCGCCGCCTTCC	Complementation of *sven0913*
Compl13_BamHI_R	CGGTGGATCCTCAGGCCGGGCCCGCGTCCG	Complementation of *sven0913*
Compl14_NdeI_F	GGGCGAACATATGACGCCGACGCTCCTGAC	Complementation of *sven0913*
Compl14_SacII_R	CCTCCGCGGCTACCGCCGCCCCGCCGTAG	Complementation of *sven0913*
Compl15_SpeI_F	CGAAACTAGTGAAAAAGCTCCAACTACATC	Complementation of *sven0913*
Compl15_BamHI_R	AGCGGGATCCTCACCGCTTCGCCCCCGACG	Complementation of *sven0913*
F0913_OE_NdeI	AAATATACATATGTCCACGATTTCGGATC	Overexpression of *sven0913*
R0913_OE_HindIII	AAAAGCTTTCAGGCCGGGCCCGCGTCC	Overexpression of *sven0913*
Cm_0912_RT_F	CACCGGTCCTGCGTGTCA	RT-PCR *sven0912*
Cm_0912_RT_R	TCGCCCATCCCTCGAAAG	RT-PCR *sven0912*
Cm_0913_RT_F	GTCATCGACGGCATGCAC	RT-PCR *sven0913*
Cm_0913_RT_R	GTACGTAATATCCGCAGCGC	RT-PCR *sven0913*
Cm_0915_RT_F	ATGACGACCCACCAGACAG	RT-PCR *sven0915*
Cm_0915_RT_R	CCGACGAGGAACATGAACAG	RT-PCR *sven0915*
Cm_0916_RT_F	GTCGGAATTCATGCTGTCCG	RT-PCR *sven0916*
Cm_0916_RT_R	GAGGAGGAACGTGATGAGGA	RT-PCR *sven0916*
Cm_0920_RT_F	GTACGAGATCTGCCTGACCA	RT-PCR *sven0920*
Cm_0920_RT_R	CTTGATGGGCTTGGACTCGA	RT-PCR *sven0920*
Cm_0924_RT_F	TCTCCATCAACGCCTACCTC	RT-PCR *sven0924*
Cm_0924_RT_R	GACCTGCTTCATCATCTCGC	RT-PCR *sven0924*
Cm_0927_RT_F	CTGCGAAGGTTCGAGGACT	RT-PCR *sven0927*
Cm_0927_RT_R	CACCGAGTCCAGCGAGTAGT	RT-PCR *sven0927*
Cm_0929_RT_F	GACCACATCGACCTCTGCTA	RT-PCR *sven0929*
Cm_0929_RT_R	AGGGAGTACTCGGACTGGAC	RT-PCR *sven0929*
Cm_0930_RT_F	GCATCACCGAGGAACAGC	RT-PCR *sven0930*
Cm_0930_RT_R	CCAGTCGAAGTACTCCACCA	RT-PCR s*ven0930*
